# Hierarchical chromatin organization detected by TADpole

**DOI:** 10.1093/nar/gkaa087

**Published:** 2020-02-21

**Authors:** Paula Soler-Vila, Pol Cuscó, Irene Farabella, Marco Di Stefano, Marc A Marti-Renom

**Affiliations:** 1 CNAG-CRG, Centre for Genomic Regulation (CRG), Barcelona Institute of Science and Technology (BIST), Barcelona 08028, Spain; 2 Gastrointestinal and Endocrine Tumors Group, Vall d’Hebron Institute of Oncology (VHIO), Barcelona 08035, Spain; 3 Centre for Genomic Regulation (CRG), Barcelona Institute of Science and Technology (BIST), Dr. Aiguader 88, Barcelona 08003, Spain; 4 Universitat Pompeu Fabra (UPF), Pg. Lluis Companys 23, Barcelona 08003, Spain; 5 ICREA, Pg. Lluis Companys 23, Barcelona 08010, Spain

## Abstract

The rapid development of Chromosome Conformation Capture (3C-based techniques), as well as imaging together with bioinformatics analyses, has been fundamental for unveiling that chromosomes are organized into the so-called topologically associating domains or TADs. While TADs appear as nested patterns in the 3C-based interaction matrices, the vast majority of available TAD callers are based on the hypothesis that TADs are individual and unrelated chromatin structures. Here we introduce TADpole, a computational tool designed to identify and analyze the entire hierarchy of TADs in intra-chromosomal interaction matrices. TADpole combines principal component analysis and constrained hierarchical clustering to provide a set of significant hierarchical chromatin levels in a genomic region of interest. TADpole is robust to data resolution, normalization strategy and sequencing depth. Domain borders defined by TADpole are enriched in main architectural proteins (CTCF and cohesin complex subunits) and in the histone mark H3K4me3, while their domain bodies, depending on their activation-state, are enriched in either H3K36me3 or H3K27me3, highlighting that TADpole is able to distinguish functional TAD units. Additionally, we demonstrate that TADpole's hierarchical annotation, together with the new DiffT score, allows for detecting significant topological differences on Capture Hi-C maps between wild-type and genetically engineered mouse.

## INTRODUCTION

The organization of the genome in the nucleus has been shown to play a prominent role in the function of the cell. Increasing evidence indicates that genome architecture regulates gene transcription (1,2), with implications on cell-fate decisions (3–5), development (6) and diseases such as developmental abnormalities (7,8) and neoplastic transformations (9–11).

Genome organization is characterized by complex and hierarchical layers (1). For example, fluorescence *in-situ* hybridization revealed that chromosomes are positioned in preferential areas of the nucleus called chromosome territories (12). This large-scale feature has been confirmed by high-throughput Chromosome Conformation Capture (Hi-C) experiments (13), that provide a genome-wide picture in which inter-chromosomal interactions are depleted relative to intra-chromosomal ones. Analysis of Hi-C data also revealed the segregation of the genome into multi-megabase compartments characterized by different GC-content, gene density and diverse chromatin marks (13–15). Microscopy approaches, in spite of considerable variability, have corroborated the spatial segregation of such compartments at the single-cell level (16). At the sub-megabase level, Hi-C experiments also revealed the presence, validated by microscopy approaches (17–19), of self-interacting regions termed topologically associating domains (TADs) (20,21). TADs are composed of dense chromatin interactions that promote 3D spatial proximity between genomic *loci* that are distant in the linear genome sequence. Since many of these interacting *loci* are cis-regulatory elements, TADs are usually considered as the structural and functional units of the genome that define the regulatory landscape (22,23) conserved across cell types and species (20,24). Moreover, TADs boundaries are often demarcated by housekeeping genes, transcriptional start sites and specific chromatin insulators proteins, such as CCCTC-binding factor (CTCF) and cohesin protein complex (20,25). TADs appear to be further organized in a hierarchical fashion. For example, in mammalian cells, concepts such as ‘metaTADs’ (26) or ‘sub-TADs’ (27) have been introduced. The former is used to define a superior hierarchy of domains-within-domains that are modulated during cell differentiation, while the latter is used to emphasize how and where the *cis*-regulatory elements establish physical interactions that contribute to gene regulation.

Several computational methods to identify and characterize TADs from Hi-C interaction data have been compared (28,29). Based on different assumptions about how TADs are divided, these methods can be broadly classified as disjointed or overlapping. The former methods consider TADs as individual and unrelated structures with no possible mutual intersections (e.g. directionality index (DI) (20), insulation score (IS) (30), ClusterTAD (31), ICFinder (32)). The latter methods assume that TADs are overlapping and related structures with shared content. However, only a few algorithms (such as Arrowhead (33), Armatus (34), 3DNetMod (35) TADtree (36), CaTCH (37), GMAP (38), Matryoshka (39) and PSYCHIC (40)) can identify nested domains where each domain contains other sub-domains, profiling a hierarchical chromatin architecture.

Here, we present TADpole, a bioinformatics tool to disentangle the full structural chromatin hierarchy. Notably, TADpole is robust both at technical and biological benchmarks based on a published study (29) and does not rely on mandatory parameters. We prove the effectiveness of TADpole investigating the inherent chromatin hierarchy in Capture Hi-C data (cHi-C) (41), where chromosome topology is altered with local genomic inversions that drive gene misexpression associated to congenital malformations in mouse (42).

## MATERIALS AND METHODS

### The TADpole pipeline

**Figure 1.**
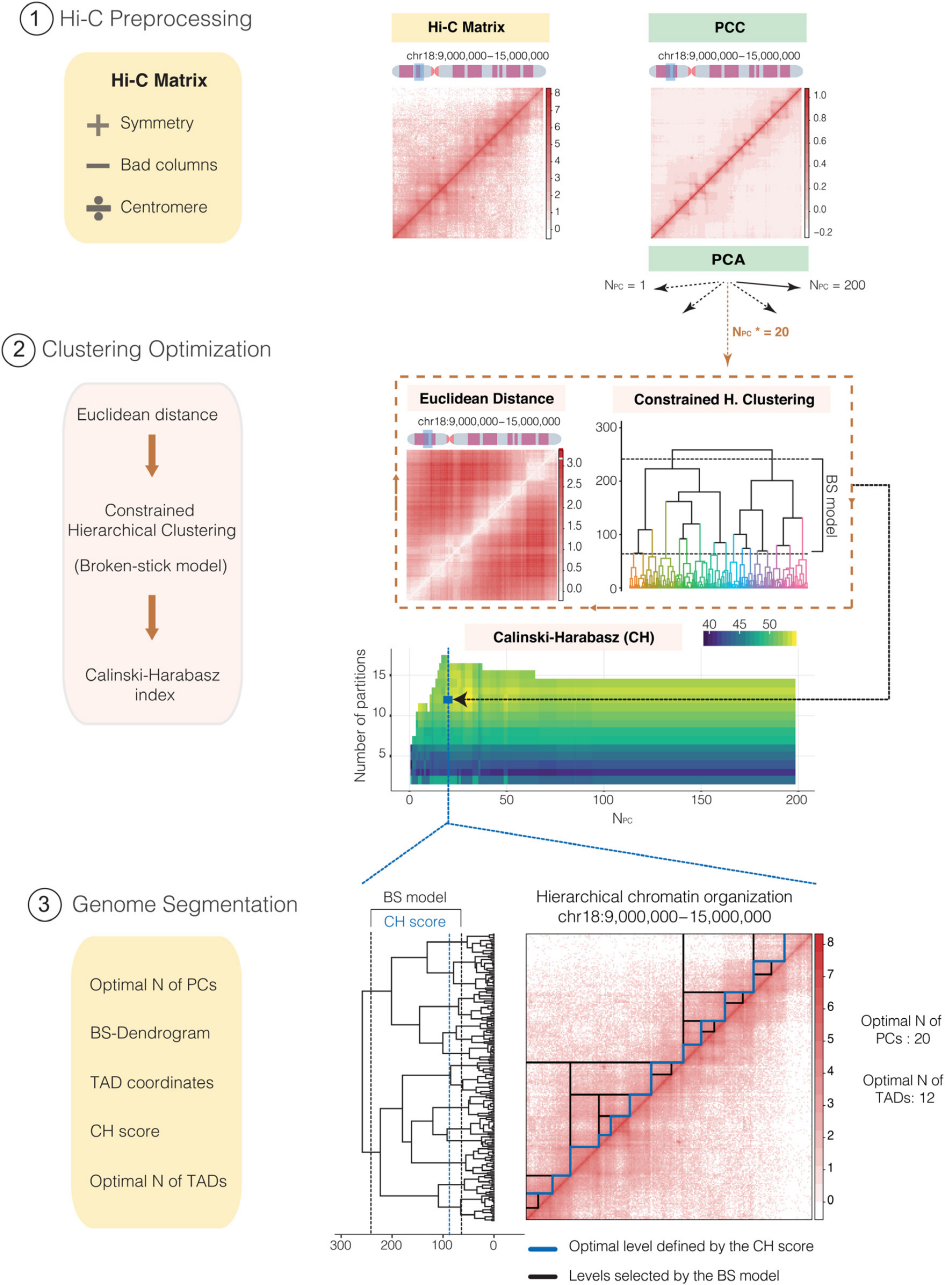
General overview of TADpole tool. Schematic overview of the TADpole algorithm. (1) TADpole input is an all-versus-all tab-limited Hi-C matrix. The matrix is checked for symmetry and low-quality columns (called as bad columns—BC) are removed. Large matrices of entire chromosomes are optionally split at the centromere to create two smaller sub-matrices corresponding to the chromosomal arms. Next, matrix denoising and dimensionality reduction take place by computing the corresponding PCC matrix, and by performing a PCA on it. (2) Per each number of first PCs retained (from 1 to 200), the corresponding PC matrix is transformed into its Euclidean distance matrix (EDM). The EDM serves as the input to perform the constrained hierarchical clustering (CH-clust). The range of significant hierarchical levels is fixed from level 1 (corresponding to partitioning the region into 2 TADs) up to an upper bound given by the broken-stick model (BS), then the Calinski-Harabasz (CH) index is used to select the optimal level. (3) As output, TADpole returns the optimal number of first PCs (N_pc_*) retained to obtain the optimal set of TADs, the dendrogram with the significant hierarchical levels, the coordinates of the chromatin domains for each level with its associated CH index, and the optimal number of TADs. A real example of TADpole tool applied to a 6Mb-region (chr18:9,000,000–15,000,000) of a human Hi-C dataset (HIC003; SRR1658572) at 30 kb resolution obtained from Rao *et al.* (15). Two bad columns were detected and removed from the input data and then, the PCC and the PCA were computed (using the first 200 PCs). Using the first 20 PCs, the EDM is computed and is used as the input for the CH-clust. A total of 16 hierarchical levels are retrieved according to the BS model and, for each one, the CH index is computed (this process is repeated iteratively for each set of PCs analyzed). This step produces a matrix of CH indexes (with the result of the 200 computed dendrograms) from which the highest average score is selected (highlighted with the blue square), in this case corresponding to 12 TADs and the first 20 PCs (N_pc_*). Taking these values, a complete dendrogram of the Hi-C matrix is retrieved, cut using the broken-stick model to select significant levels (containing from 2 to 17 TADs, shown between black lines) and, from them, the highest-scoring level according to the CH index is selected (blue line). On the right, the Hi-C contact map is presented showing the complete hierarchy of the significant levels selected by the BS model (black lines) along with the optimal one in 12 specific TADs, as identified by the highest CH index (blue line).

TADpole performs three main steps (Figure 1): (i) preprocessing of the input Hi-C dataset, (ii) constrained hierarchical clustering optimization and (iii) genome segmentation. TADpole has been implemented as an R package available at https://github.com/3DGenomes/TADpole.

#### Preprocessing of the input dataset

TADpole is designed to process all-versus-all intra-chromosomal interactions matrices representing an entire chromosome, or a continuous chromosome region. The input is a generic tab-separated file containing the interaction matrix (*M*) with N rows and N columns, where N is the number of bins in which the chromosome region is divided. Each position of the matrix (*M_ij_*) contains the number of interactions (raw or normalized) between the corresponding pair of genomic bins *i* and *j*. An additional filtering step can be applied to exclude columns (and the corresponding rows) with a low number of interactions (called as bad columns), which typically arise from local biases (43). Specifically, the columns that contain an empty cell at the main diagonal, and those whose cumulative interactions are below the first (by default) percentile, are excluded from the analysis. To enhance the signal-to-noise ratio, the interaction matrix is transformed into its Pearson correlation coefficient (PCC) matrix (13), and principal component analysis (PCA) is performed on it using the *prcomp* function from the *stats* R package (44). Only the first 200 (by default) principal components (N_PC_) are retained, which are enough to extract more than 85% of the variance in the test datasets (Supplementary Figure S1). To reduce memory usage and processing time, TADpole has the option to divide the interaction matrix by the centromere (considered to be the longest contiguous stretch of columns with no interactions in the Hi-C matrix) and process each chromosomal arm separately. This option is particularly recommended when working with matrices of more than 15 000 bins.

#### Constrained hierarchical clustering optimization

Per each set of first PCs, N_PCs_, the dimensionally-reduced matrix is transformed into a Euclidean distance matrix. This distance matrix is then partitioned into topological domains using a constrained hierarchical clustering procedure as implemented in the Constrained Incremental Sums of Squares clustering method (*coniss*) of the *rioja* R package (45). This analysis explicitly assumes the following two priors: first, the genome is organized in a hierarchical manner, with higher-order structures containing lower-order ones, and second, every pair of contiguous genomic loci must either belong to the same self-interacting domain or to the immediately contiguous one. Thus, the constrained hierarchical clustering results in a tree-like description of the genome organization. Next, using the broken-stick model as implemented in the *bstick* function from the *rioja* R package (45), the dendrogram is cut at a maximum significant number of levels (max(N_D_)). The Calinski-Harabasz (CH) index is then computed for each dendrogram (from 1 to 200, corresponding to each set of N_PCs_ computed) and for each significant level (from 2 to max(N_D_)) using the *calinhara* function from the *fpc* R package (46). The dendrogram with the highest average CH index is selected, and the level with the maximum CH index of that dendrogram is taken as the optimal. This analysis jointly identifies an optimal number of first principal components (N_PCs_*) and an optimal number of TADs (N_D_*).

#### Genome Segmentation

TADpole generates four main descriptors that recapitulate the entire sets of results, namely: (i) the optimal number of principal components (N_PCs_*) (ii) the cut dendrogram at the maximum significant number of levels identified by the broken stick model (max(N_D_)); (iii) the start and end coordinates of all the TADs identified for each hierarchical level and the CH index associated to it; (iv) and the optimal number of TADs (that is, the optimal level plus 1). All the TADpole output is organized in a comprehensive R object.

### TADpole benchmark analysis

#### Benchmark Hi-C dataset and scripts

**Figure 2.**
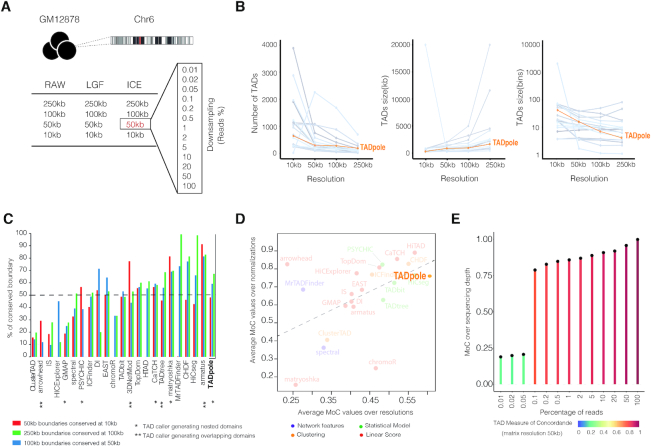
Technical benchmarking of TADpole. (**A**) General overview of the dataset used for the technical benchmarking analysis from Zufferey *et al.* (29). The dataset includes the Hi-C interaction matrix of chromosome 6 in GM12878 cells in 24 different forms (‘Materials and Methods’ section). (**B**–**E**) Results of the technical benchmarking of TADpole considering the optimal level with its corresponding TADs in each case and comparing with the other 22 TAD callers considered in Zufferey *et al.* (29). (B) The number, and the size of TADs in kilobases (kb) and number of bins. Each gray line represents each one of the other 22 TAD callers. (C) Percentage of conserved TADs boundaries over different resolutions across the 22 TAD callers. (D) The average MoC values across normalization strategies against the average MoC values across resolutions. Colors group the different TAD callers according to their specific mathematical approach. (E) MoC values for different down-sampling matrices from the ICE-normalized interaction matrix at 50kb of resolution. Panels B–D have been adapted from Figures 2C, 1D-E and Supplementary S2B of Zufferey *et al.* (29) to include TADpole in the comparison of TAD callers.

A pre-existing benchmark dataset, that comprises Hi-C interaction matrices of the entire chromosome 6 in the human cell line GM12878, was used for the analysis (29). A total of 24 different conditions were tested: (i) twelve matrices given by the combination of four different resolutions (10, 50, 100 and 250 kb) and two normalization strategies (Iterative Correction and Eigenvector decomposition (ICE) (14) and parametric model of Local Genomic Feature (LGF) (47)) together with the raw data, and (ii) twelve matrices obtained by down-sampling of the ICE interaction matrix at 50 kb resolution (Figure 2A). The scripts for benchmarking were downloaded and used as released in the repository https://github.com/CSOgroup/TAD-benchmarking-scripts (29) (‘Data Availability’ section). The processed Hi-C dataset was shared by Zufferey and colleagues, thus eliminating from the analysis possible biases associated with the use of different pipelines for Hi-C interaction data reconstruction (28). To compare on equal footing with the other 22 TAD callers in the same benchmark, only hierarchical levels that comprise at least 10 chromatin domains were taken into consideration for the analysis. Within these levels, the optimal one was identified using the CH index as described before.

#### The technical benchmark

The optimal TAD divisions given by TADpole were compared over different resolutions, normalization strategies, and sequencing depths, as previously described (29). To study the degree of TAD borders conservation, two different metrics were applied:

- The overlap score (29) was used to compare chromatin domains annotated across different resolutions. This is the percentage of overlapping borders, with one bin of tolerance. The statistical significance of each overlap score was estimated by drawing 10 000 random chromatin partitions at the finer resolution (preserving the optimal number of TADs annotated in the real case) and computing their overlap with the subdivision at the coarser resolution. The *P*-value of the real-case was computed as the fraction of randomized partitions with larger overlap.
- The Measure of Concordance (MoC) (29) was used to quantitatively express the extension of the agreement among two partitions in TADs across different resolutions and normalization strategies. The MoC is defined by:
  $$\begin{eqnarray*}&&MoC\ \left( {P,Q} \right)\\ &&\quad = \left\{ {\begin{array}{cll} 1\!\!\!&, & \quad {if\ {N_P} = {N_Q} = 1}\\ {\frac{1}{{\left( {\sqrt {{N_P}{N_Q}} - 1} \right)}}\left( {\mathop \sum \limits_{i = 1}^{{N_P}} \mathop \sum \limits_{j = 1}^{{N_Q}} \frac{{{F_{ij}}^2}}{{{P_i}{Q_j}}} - 1} \right)}\!\!\!&, &\quad {\rm otherwise} \end{array}} \right. \end{eqnarray*}$$

where, P and Q are the partitions under comparison constituted by N_P_ and N_Q_ TADs, respectively. P_i_ and Q_j_ are two individual TADs within P and Q of size (in base-pairs) ||P_i_|| and ||Q_j_||. At last, ||F_ij_|| is the size (in base-pairs) of the overlapping between the two TADs P_i_ and Q_j_. The MoC ranges from 0 for discordant partitions to 1 for completely identical ones (48).

We also assessed the computational performance of TADpole using two metrics: the execution time and the maximum memory usage. The former was computed using the *microbenchmark* R package (49) and the latter was calculated using the *memtest.sh* script from https://github.com/rcortini/sesame-manuscript repository. This performance analysis was run on Intel (R) Xeon (R) CPU E5-2660 v2 @ 2.20GHZ with 512Gb of RAM.

#### The biological benchmark

To test the biological relevance of the TADs identified by TADpole, we studied: (i) their association with the main chromatin architectural proteins (CTCF, SMC3 and RAD21) and two histone modifications (H3K4me3 and H3K9me3) at TAD borders, and (ii) two other histone modifications (H3K27me3 and H3K36me3) in TAD bodies. The ChIP-seq data of these chromatin features were downloaded from ENCODE (https://www.encodeproject.org/) (50) (Supplementary Table S1). For architectural protein with more than one replica (CTCF and RAD21), we analyzed: (i) the profile of individual replicas, (ii) the cumulative profile, which is the union of all called peaks and (iii) the consensus profile, which is the intersection of the peaks identified in all replicas as determined by the *multiIntersectBed* function of the BEDTools suite (51). To analyze the co-occupancy of the architectural proteins, we first computed a consensus cohesin complex profile between the cumulative profile of RAD21 and the individual experiment of SMC3. The resulting consensus cohesin complex profile was intersected with the cumulative profile of CTCF. For the analysis of the histone modification H3K4me3, we used the file containing the replicated peaks. For H3K9me3, since such peaks were not available for download, we generated them by first pooling the aligned reads of the different replicas, for both the target and control samples, and subsequently running MACS2 (52) on the two resulting files with the option *–broad* set. For H3K27me3 and H3K36me3, we used the fold change over control pooled replicas as in Zufferey *et al.* (29).

**Figure 3.**
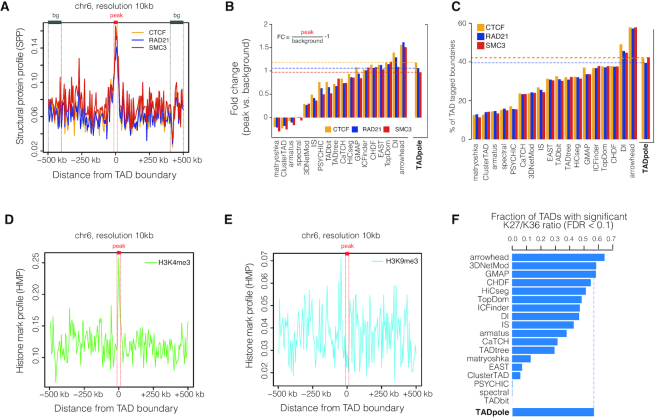
Biological benchmarking of TADpole. TAD boundaries used in these analyzes are the result of the optimal TAD partition defined by TADpole of the Hi-C matrix at 10 kb of resolution. (**A**) SPP around the TAD boundaries (peak region in red, background region in gray) are shown for the consensus profile of CTCF, RAD21 and SMC3. (**B**) Fold-change of the SPP of CTCF, RAD21 and SMC3 at TAD boundaries for all the 22 TAD callers. (**C**) Percentage of TAD boundaries hosting CTCF, RAD21 and SMC3 for all the TAD callers. (**D** and**E**) HMP computed around TAD boundaries for active promoter mark (H3K4me3) and repressive histone mark (H3K9me3). (**F**) The fraction of TADs with significant log10 ratio between H3K27me3 and H3K36me3 obtained for TADpole and for other 22 TAD callers. Panels B, C and F have been adapted from Figure 5D, E, I from Zufferey *et al.* (29).

Mirroring the approach in Zufferey *et al.* (29), four different metrics were computed:

- *Structural protein (or histone mark) profile from ChIP-seq peaks around TAD boundaries*. Per each ChIP-seq experiment considered in the analysis, a structural protein profile (SPP) or a histone mark profile (HMP) was calculated. The SPP or the HMP were defined as the average number of peaks over 5 kb intervals inside a 1 Mb region around each TAD boundary (±500 kb). A consistent enrichment at the TAD boundary results in a SPP or HMP that peaks at around 0 kb (the location of the TAD boundary). A SPP is shown, for instance, in Figure 3A, and an HMP in Figure 3D and E.
- *Fold change of* *a SPP at the TADs boundary*. The fold change was computed as the ratio between average SPP in a region around the TAD boundaries (0 kb ±1 bin) over the SPP in a region of 100 kb located 400 kb apart from the TAD boundaries. This fold change is decreased by one so that an absence of enrichment (ratio = 1) is represented by zero.
- *Ratio of TAD boundaries hosting a specific protein*. The ratio was computed as the number of TAD boundaries that harbor at least one ChIP-seq peak (±1 bin) over the total number of boundaries.
- *Ratio of ChIP-seq signals in TAD bodies*. The ChIP-seq signal (fold change over control) was coarse-grained in bins spanning the 10% of the average TADs size. In each bin, the log10 ratio between the H3K27me3 and H3K36me3 signals was calculated. A shuffle test was used to compute an empirical *P*-value per bin. Next, the Benjamini-Hochberg procedure was applied to adjust the *P*-values in each bin of a TAD and to assign a false discovery rate (FDR) per TAD. The fraction of TADs having an FDR < 0.1 was reported in the final barplot (see for instance Figure 3F).

### Difference score between topological partitions (DiffT)

To compare pairs of topological partitions, P and Q, identified by TADpole at a fixed level of the hierarchy, we defined a difference topology score (DiffT). Specifically, the partitioned matrices were transformed into binary forms p for P, and analogously q for Q, in which each entry p_ij_ (q_ij_) is equal to 1 if the bins *i* and *j* are in the same TAD and 0 otherwise. Then, DiffT is computed as the normalized (from 0 to 1) difference between the binarized matrices as a function of the bin index *b* as:

$$\begin{equation*}DiffT\ \left( b \right) = \frac{{\mathop \sum \nolimits_{i = 1}^b \mathop \sum \nolimits_{j = 1}^N \left| {{p_{ij}} - {q_{ij}}} \right|}}{{\mathop \sum \nolimits_{i = 1}^N \mathop \sum \nolimits_{j = 1}^N \left| {{p_{ij}} - {q_{ij}}} \right|}}\ \end{equation*}$$

where *N* is the total number of bins. The calculation of DiffT is illustrated in Supplementary Video S1.

To test whether the identified TADs partition in Q is significantly different from P at each level of the chromatin hierarchy, a statistical analysis was introduced. This analysis assesses the significance of DiffT at each bin of the matrix. A total of 10 000 random partitions of the *locus* were simulated, excluding the bad columns of the Q matrix (see below). The DiffT score was computed between simulated and P partitions (DiffT_simulated-p_). At each bin, the fraction of DiffT_simulated-p_ lower or equal to the DiffT_q-p_ score estimates the *P*-value. A *P*-value < 0.05 means that a significant amount of the total DiffT score is located in the genomic region starting at the bin under consideration onward. Hence, the bin(s) with the minimum *P*-value marks the starting point of the *locus* where the most significant fraction of the DiffT score is located.

The DiffT score analysis was used to compare the TADpole partitions in two Capture Hi-C (cHi-C) experiments designed by Kraft and colleagues over the genomic interval chr1: 71 000 000–81 000 000 in embryonic day E11.5 mouse cells using mm9 as a reference mouse assembly (42). Specifically, the two homozygous strains were considered comprising the wild-type (WT) and the so-called inversion1 (Inv1). The normalized cHi-C interaction maps were downloaded from GEO (53) at the GSM3261968 (WT) and GSM3261969 (Inv1) entries. Next, the gene-dense region (chr1: 73 920 000–75 860 000) was extracted and used for DiffT analysis.

To compare the TADpole results on this cHi-C data, the DiffT score analysis was applied also on the TADs partitions identified by other 8 hierarchical TAD callers on the same targeted *locus*. The parameters used and the process of selecting the hierarchical levels are specified in Supplementary Table S2.

## RESULTS

### TADpole benchmark analysis

To quantitatively compare TADpole with other 22 TAD callers, we applied the multiple conditions test proposed in Zufferey *et al.* (29) on the same reference benchmark dataset (Figure 2A and ‘Materials and Methods’ section).

#### Technical benchmarking

We assessed various technical aspects of TADpole as well as the robustness of TADpole in identifying domains at different resolutions, normalization strategies, and sequencing depths of the input matrix (Figure 2A). First, we examined the number and the size (in kilobases and in bins) of the optimal number of TADs identified in the ICE-normalized maps at different resolutions (Figure 2B). We found that, as the resolution of the Hi-C interaction map decreased, the number of TADs and the mean TAD size in bins decreased with a 4-fold reduction. TADpole followed a similar trend (positive when TADs are measured in kilobases and negative when TADs are measured in bins) as the majority of the other TAD callers independently of the applied normalization strategy (Supplementary Table S3).

We also inspected if TADpole identified robust boundaries over different resolutions. To measure the degree of conservation, we tested if a border detected in the ICE normalized Hi-C matrix at a certain resolution was conserved in the resolution immediately finer (Figure 2C and Supplementary Figure S2). Overall, TADpole conservation test ranked 6–7^th^ over the 22 TAD callers. Specifically, at the coarser resolutions, that is 250 kb versus 100 kb, we found a high agreement (67%), that decreased only slightly to (59%) at intermediate ones (100 versus 50 kb). Interestingly, we found that even at the finer resolutions (50 kb versus 10 kb), where the 48% of the borders were conserved, this analysis was consistent with a statistically significant overlap (*P*-value < 0.05).

Next, we used the MoC (‘Materials and Methods’ section) to estimate if the number and the position of the borders of TADs identified by TADpole were affected by the matrix resolution and by normalization strategy. Interestingly, we found that the MoC over different matrix resolutions had values in the [0.45,0.82] range with an average MoC of 0.63, and ranked first when compared with the other 22 TAD callers previously benchmarked (29). TADpole was also robust over different normalization strategies with an average MoC of 0.74, ranking ninth over the 22 TAD callers. Comparing the average of resolutions versus normalizations MoC values of TADpole with the rest of TAD callers (Figure 2D), we found that TADpole appeared in the top-right corner of the plot demonstrating its overall high robustness and confidence to identify optimal chromatin domains independently of the resolution and the normalization strategy applied to the input Hi-C matrix. We also tested the TADpole propensity to identify consistent optimal chromatin domains independently of the sequencing depth (Figure 2E). We compared the TADs obtained by doing 12 different sub-samplings of the ICE-normalized interaction matrix at 50 kb of resolution against the full interaction matrix using the MoC. We found that TADs defined by TADpole were clearly robust to down-sampling with a MoC score of 0.79 with just 0.1% of the total data. This feature classified TADpole as the top TAD caller with respect to the other 22 tools.

#### Computational performance

We also compared the execution time of TADpole with the other 22 TAD callers across different resolutions (1000, 250, 100, 50, 10 kb). TADpole performs as good as the bulk of the TAD callers. (Supplementary Figure S3). The maximum memory usage was computed for one normalization dataset (LGF) and is mostly quadratic to the number of bins in the Hi-C matrix (Supplementary Figure S3).

#### Biological benchmarking

Due to the lack of a gold standard to define TADs in Hi-C interaction maps (28,29), we investigated the biological relevance of the domains identified by TADpole in terms of their association with biological features that have been shown to have an important role in the formation and maintenance of TADs. After computing the SPP for the intersection of the peaks in CTCF and RAD21 replicate experiments, and SMC3 experiment, we found an enrichment at TAD boundaries (Figure 3A). To compare TADpole with the other TAD callers, we computed the fold change enrichments at the domain borders with respect to the flanking regions (Figure 3B). TADpole resulted in a fold change enrichment around 1 for each of the three main architectural proteins (1.18 in CTCF, 1.06 in RAD21 and 0.97 in SMC3, respectively), that was consistent with a significantly high peak at the border compared with the background (*P*-value < 10^−5^). In this analysis, TADpole ranked as the sixth TAD caller. Additionally, we quantified the percentage of boundaries that are occupied by CTCF or cohesin complex subunits individually, as well as the percentage of boundaries in which these two architectural proteins co-occur. Notably, more than 40% of the TADs boundaries contain at least one of the three main architectural proteins analyzed, being CTCF (42%) and SMC3 (42%) the most represented. In this analysis, TADpole ranked third within the set of 22 TAD callers (Figure 3C). Additionally, 36.6% of the TAD boundaries analyzed had both major subunits of the cohesin complex; while a 36.2% of them were occupied by both cohesin subunits in association with CTCF sites.

To assess how this validation can be affected by biological variability over different experiments, we repeated the same analysis on each individual replicate as well as their union (‘Materials and Methods’ section) for CTCF and RAD21 (Supplementary Figure S4). In all the cases, a peak of the SPP was consistently found at TAD boundaries (Supplementary Figure S4A), with fold change values from 0.97 to 1.09 for individual replicates and 1.05 for the cumulative profile of CTCF and from 0.87 to 0.97 for individual replicates and 0.91 for the cumulative profile of RAD21 (Supplementary Figure S4B). The percentage of occupied boundaries by CTCF varied from 47 to 51% for individual replicas and 54% for the union profile and from 41 to 48% for individual replicas and 49% for the union profile of RAD21 (Supplementary Figure S4C). These results demonstrate that biological variability over different experiments only marginally impacts on the biological benchmarking of TADpole.

We also studied whether the TADpole boundaries are enriched for active (H3K4me3) or inactive (H3K9me3) histone marks by computing the respective HMP (see ‘Materials and Methods’ section). As expected, the HMP of H3K4me3 was sharply peaked at the TAD boundaries (Figure 3D), while the HMP of H3K9me3 resulted in the absence of enrichment (Figure 3E) (15,20,54). As regulons, TADs are expected to be either transcriptionally active or inactive (15) with the TAD body enriched in active or inactive histone mark. To assess whether the TADpole TADs follow this observation, we considered the ChIP-seq signals of two histone marks: H3K36me3 for transcriptional activity, and H3K27me3 for repression; then we measured the fraction of TADs where the log10 of their ratio (H3K27me3/H3K36me3) was significantly higher (enrichment in repressive mark) or lower (enrichment in active mark) than zero (‘Materials and Methods’ section). Notably, we found that the majority (57%) of the TADs identified by TADpole have a defined active or inactive state, positioning TADpole within the top four TAD callers based on this criterion (Figure 3F).

### Applications to capture Hi-C datasets

**Figure 4.**
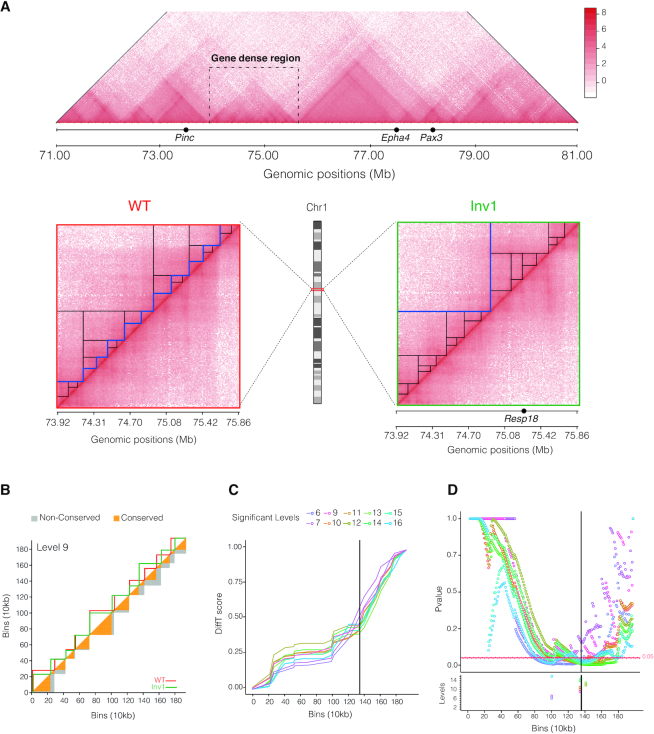
Characterization of topological difference in capture Hi-C datasets. (**A**) Top: Overview of the entire WT captured region (chr1:71.00–81.00Mb) in Kraft *et al.* (42). The gene-dense region harbors the breakpoint of the inversion (Inv1). Bottom: Capture Hi-C maps of the gene-dense region (chr1:73 920 000–75 860 000) in WT and inversion 1 (Inv1) strains. In both matrices, the significant hierarchical levels are shown as black lines and the optimal one as a blue line. (**B**) Example of DiffT score for the ninth hierarchical level (corresponding to 10 TADs) of the dendrogram. The upper triangle of the matrix shows the TADs identified by TADpole in WT and Inv1 matrices as red and green continuous lines, respectively. The lower triangle of the matrix shows the conserved (in orange) and non-conserved (in gray) areas of the TADs. In the panels **C** and **D**, the Inv1 breakpoint is highlighted with a solid black line, and only the levels that contain at least one bin with a DiffT-score associated *P*-value < 0.05, are shown. (C) DiffT score profiles as a function of the matrix bins. The calculation of the DiffT score is used to obtain a curve in panel C from the TAD partition in panel B is illustrated in Supplementary Video S1. (D) *P*-value profiles per bin for automated detection of significant differences. In the lower panel, the bins associated with minimum *P*-values per level are marked with empty dots.

The strength of TADpole is given by its capability to disentangle the entire hierarchical TAD organization from an interaction map. Here, to test its usability, we partitioned into domains a series of high-resolution interaction maps of embryonic day E11.5 mouse limb buds (42). The interaction matrices from Capture Hi-C experiments focused on a specific region (chr1:71 000 000–81 000 000) containing the *Epha* locus and several developmentally important genes such as *Pax3*, *Epha4* and *Pinc*. A series of 4 inversions were done in a specific gene-dense region, located between the *Epha4* and *Pinc* genes (chr1: 73 920 000–75 860 000). The goal of the work was to investigate the pathogenic consequences of balanced chromosomal rearrangements but the interpretation of the gene-dense region, that did not show a clear topological structure, had proven challenging (42) (Figure 4A). To show the usability of TADpole in structural comparative studies, here, we compare the WT strain (Figure 4A, left) with the sole inversion producing a homozygous strain (hereafter called Inv1), that is located between the telomeric site of *Epha4* enhancer cluster and the promoter of *Resp18* (breakpoint at chr1:75 275 966–75 898 706) (Figure 4A, right).

The hierarchical analysis of TADpole revealed the existence of 17 levels in WT (21 optimal PCs and 11 optimal TADs) and 16 levels in Inv1 (25 optimal PCs and 2 optimal TADs) (Figure 4A). After visual inspection, the TADs defined in WT and Inv1 had several differences. These involved many of the hierarchical levels and tended to accumulate in the region where the inversion was produced. To statistically quantify and localize the significant topological differences between the WT and Inv1 matrices, we computed their DiffT score profiles between the TAD at each hierarchical level (‘Materials and Methods’ section, Figure 4B and Supplementary Figure S5). We found that the DiffT profiles sharply increased close to the point of the inversion (Figure 4C). Based on the *P*-value profiles (Figure 4D), we identified two regions where the minimum *P*-values, calculated at each hierarchical level, accumulated. Notably, 70% of minimum *P*-values were located within a region, spanning 50 kb, from the point where inversion was induced, suggesting that the significant topological changes between WT and Inv1 accumulated in the inverted region.

Next, we assessed if the significant topological differences detected by TADpole could also be retrieved with other TAD callers providing hierarchical chromatin domains. 8 different tools were used: Armatus, Arrowhead, Matryoshka, TADtree, CaTCH, GMAP, PSYCHIC and 3DNetMod (Supplementary Table S2). Interestingly, TADpole was the sole TAD caller to identify the insertion point of the Inv1 mutation as the beginning of the most significant topologically different region (Supplementary Figure S5). Note that no panel is shown for PSYCHIC because it did not return any significant bin.

## DISCUSSION AND CONCLUSION

In this work, we introduced TADpole, a bioinformatics tool to identify hierarchical topological domains from all-versus-all interaction matrices. In line with previously introduced concepts such as metaTADs (26) and sub-TADs (27), we propose that, aside from an optimal number of TAD, there is a range of meaningful hierarchical chromatin subdivisions. TADpole characterizes the entire hierarchy of TADs while assessing the significance at various levels of organization, paving the way for accurate characterization of the nested genome topology and its biological role.

The principles behind this nested structure are not yet fully understood, and different probable scenarios need to be considered. Indeed, this hierarchical organization may arise from the variability of conformations observed within a cell population (55). However, in individual cells, globular structures delimited by variable boundaries (which preferentially reside at the CTCF and cohesin binding sites) with a similar appearance to TADs and sub-TADs have been observed (56). In fact, it has been determined that the shared boundaries between individual cells have a direct correspondence with TAD borders identified in cell populations (56,57) and after cohesin depletion, these TAD-like structures are maintained with changes in their boundary positions. Remarkably, in individual cells, these boundaries can be present at multiple genomic positions with a non-zero probability and, therefore, we cannot exclude the possibility that the hierarchical TAD organization is present as well in single cells. One can speculate that this hierarchical structure can act as a regulatory scaffold to tune a coordinated communication between cis-regulatory elements (58). We envisage that, in future studies, TADpole could be used to assess the relationship between metaTADs, TADs and sub-TADs, helping to untangle the complex genome topology by analyzing and comparing the topological organizations of chromatin in various cell types.

Prompted by this debate, dozens of methods have been developed to computationally define TADs (28,29) following two main general assumptions: either TADs are disjointed and unrelated, or overlaid/nested structures with shared content. TADpole belongs to the latter category. Other tools based on the same assumption, such as Arrowhead, Armatus, CaTCH, Matryoshka, TADtree, GMAP, PSYCHIC and 3DNetMod have been developed. However, these tools are quite different from TADpole's approach in several aspects, including the algorithm applied, the number of parameters required, and the output provided. Some of these methods are based on a linear score associated to each bin of the contact matrix (such as Arrowhead, Armatus, CaTCH and Matryoshka), or rely on statistical models of the distribution of Hi-C interaction data (such as TADtree, GMAP and PSYCHIC), or on graph theory to characterize the hierarchical nature of the chromatin as interconnected communities (such as 3DNetMod), while TADpole is the only one to adopt a strategy based on hierarchical clustering. Additionally, the majority of tools have multiple mandatory parameters that users have to define *a priori* (as in Arrowhead, PSYCHIC, TADtree or 3DNetMod) that can affect the results. A major advantage of TADpole is that, as GMAP or CaTCH, it does not require any mandatory parameters. Importantly, TADpole provides in an unsupervised manner, statistical criterion to identify the hierarchy of TADs (using the broken-stick model) as well as the optimal level of TAD partition (using the Calinski-Harabasz index). The output of other tools may require specific downstream analyses, from the manual selection of the TAD hierarchy, as in TADtree or 3DNetMod, to the tuning of *ad hoc* parameters, as the gamma value in Armatus and Matryoshka. At last, the majority of the TAD callers shared the characteristic of being completely open-access, however in some cases, a proprietary software is required (e.g. Matlab).

Another advantage of TADpole over existing TAD callers is its data preprocessing step. Indeed, the PCC transformation and the PCA application regularize the input matrix so that the specific normalization applied to the input and the sparsity of the data have little effect on identifying TADs. Previously, other architectural features of the chromatin have been already studied using PCA. The first principal component is widely used to identify the chromatin segregation into compartments (13). The second and the third PCs have been associated instead to intra-arm features mainly centromere-centromere and telomere–telomere interactions enrichment (14). Moreover, the first PCs have been used to assess the similarity between two interaction maps (14) as well as to quantify their reproducibility (59). Here we have demonstrated that there exists an optimal set of PCs capable of identifying the hierarchical structure of chromatin, extending the current application of PCA to characterize genome topology.

Here, we compared TADpole's performance with a set of other 22 TAD callers following the benchmark analysis performed by Zufferey *et al.* (29). TADpole identifies a number of TADs over different resolutions that are in agreement with other TAD callers (Figure 2B). The identified domains have an average size of 855kb, in agreement with the reported average TADs size in mammalian cells (∼900–1000 kb) (15). TADpole shows one of the largest consistencies over different normalization strategies (including also non-normalized data), resolutions and sequencing depths (Figure 2D and Supplementary Table S3). These features make TADpole potentially suitable for analyzing sparse datasets. Furthermore, the TADs reported by TADpole present a high enrichment of the main architectural proteins such as CTCF and major cohesin complex sub-units (*i.e.*, SMC3 and RAD21) (Figure 3A–C) and active histone mark (H3K4me3) at the TAD borders, as well a pronounced decrease in repressive histone mark (H3K9me3) (Figure 3D-E). These findings are in line with the close relationship between active transcription and boundary formation (15,20,54). The body of these TADs also shows an enrichment in either active (H3K36me3) or repressive (H3K27me3) histone marks (Figure 3F). This result is consistent with the fact that these marks may act as differentiators of active or repressive TADs, leading to the idea that TADs are the functional chromatin units (13,20–21,33,60). Overall, the benchmark analysis presented here (Figures 2 and 3) reveals that TADpole generally performs better than all the other nested TAD callers.

In a case study, we also provide a proof of TADpole's usability on a gene-dense region analyzing Capture Hi-C data in both a WT strain and a mutant one carrying a genomic inversion (41) (Figure 4). Notably, we found that (i) the region of interest indeed had a topological structure, (ii) there exist clear topological dissimilarities between the WT and the mutant experiments and (iii) the region, where the majority of topological differences are accumulated, matches the location of the inversion. Interestingly, TADpole is the unique TAD caller (Supplementary Figure S5) that in combination with the DiffT score is able to identify the inverted region as the one with the highest difference in topological partitions, proving that this strategy can isolate and localize within the genomic region of interest statistically significant topological dissimilarities.

In summary, TADpole combines straightforward bioinformatics analyses such as PCA and hierarchical clustering to study continuous nested hierarchical segmentation of an all-versus-all intra-chromosomal interactions matrix. Additionally, we demonstrated the technical and biological robustness of TADpole and its usability in identifying topological difference in high-resolution capture Hi-C experiments. TADpole is released as a publicly-available, open-source and numerically efficient R tool. As such, TADpole represents a comprehensive tool that fulfils the needs of the scientific community for an accurate TAD caller able to comprehensively study the interplay between the hierarchical chromatin topology and genomic function.

## DATA AVAILABILITY

The TADpole is freely available for download as an R package at https://github.com/3DGenomes/TADpole. The scripts for the technical and biological benchmarks were obtained from the repository https://github.com/CSOgroup/TADbenchmarking-scripts (28). Specifically, the script *fig2_fig3_fig4_fig5_moc_calc.R* was used for panels Figure 2B–E, the script *StructProt_EnrichBoundaries_script.R* for panels Figure 3A–D, and the script *HistMod_script.sh* for the panel in Figure 3E. Default parameters were applied.

## Supplementary Material

gkaa087_Supplemental_Files
