# Supplementary material for: Hierarchical chromatin organization detected by TADpole
Source: Nucleic Acids Res. 2020 Feb 21;48(7):e39. doi: 10.1093/nar/gkaa087 (PMC7144900; doi:10.1093/nar/gkaa087)
Supplement: gkaa087_Supplemental_Files [file gkaa087_supplemental_files.zip › 20200128_TADpole_SupFiles-.docx]

Supplementary files to:

**­Hierarchical chromatin organization detected by TADpole.**

Paula Soler-Vila^1,†^, Pol Cuscó^2,†^, Irene Farabella^1^, Marco Di Stefano^1,*^ and Marc A. Marti-Renom^1,3,4,5,*^

^1^CNAG-CRG, Centre for Genomic Regulation (CRG), Barcelona Institute of Science and Technology (BIST), Barcelona, Spain.

^2^Gastrointestinal and Endocrine Tumors Group, Vall d’Hebron Institute of Oncology (VHIO), Barcelona, Spain.

^3^Centre for Genomic Regulation (CRG), Barcelona Institute of Science and Technology (BIST), Barcelona, Spain.

^4^Universitat Pompeu Fabra (UPF), Barcelona, Spain.

^5^ICREA, Barcelona, Spain.

†Joint first authors

*To whom correspondence should be addressed. Emails: [martirenom@cnag.crg.eu](mailto:martirenom@cnag.crg.eu) & [marco.distefano@cnag.crg.eu](mailto:marco.distefano@cnag.crg.eu)


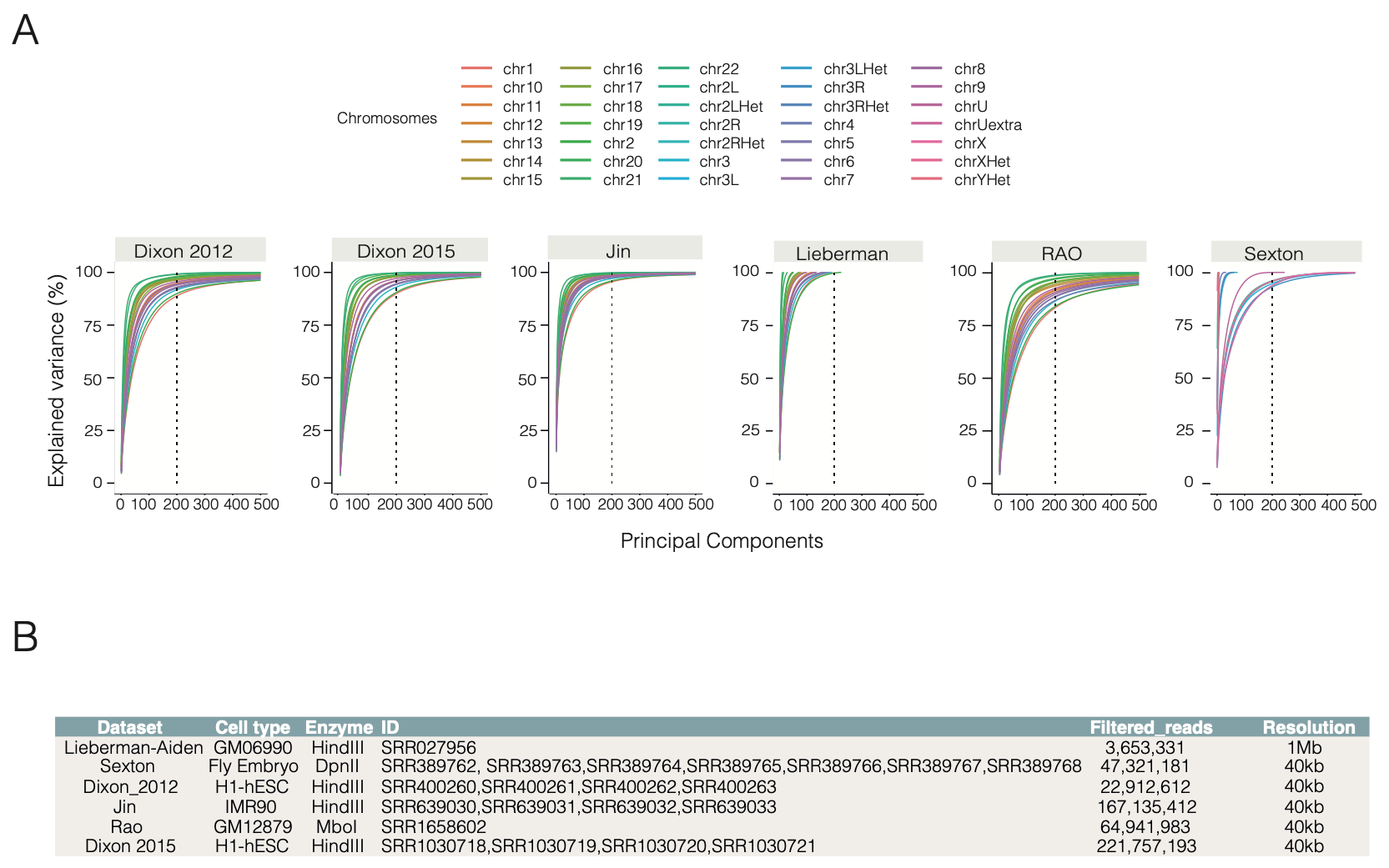


**Supplementary Figure 1. Percentage of explained variance as a function of the number of retained principal components for various datasets. (A)** Each continuous line represents a different chromosome, and the vertical dashed lines mark the default number (200) of first PCs (N_PCs_) retained by TADpole. **(B)** The six Hi-C datasets used, identify by: cell type, restriction enzyme, the NCBI accession numbers, number of the valid reads retrieved after filtering using an in-house pipeline based on TADbit (56), and binning size. Datasets with multiple NCBI entries were merged and (after filtering) the resulting matrices were binned using an equal bin-width of 40kb, with the exception of Lieberman-Aiden dataset (13) which was binned at 1Mb.


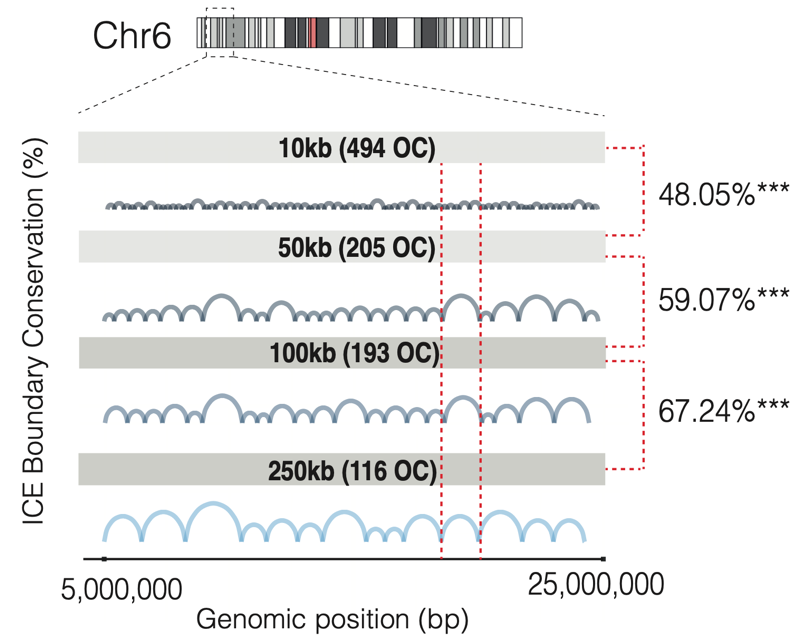


**Supplementary Figure 2**. Percentage of conserved TADs boundaries across different resolutions on the entire chromosome 6. The diagram illustrates the analysis on a random *locus* from 5 to 25Mb. The p-value is computed using a shuffle test **(Material and Methods)**.

**Supplementary Figure 3. Computational analysis of TADpole.** **(A)** Execution time (in logarithmic scale) of the all TAD callers analyzed. The average value computed between the two normalization strategies (ICE and LGF) is shown across resolutions (1000kb, 250kb, 100kb, 50kb, 10kb). **(B)** Memory usage test of TADpole. Each dot represents the maximum memory usage computed for LGF normalization matrices across different resolutions (1000kb, 250kb, 100kb, 50kb, 10kb).

**Supplementary Figure 4. Biological replicas benchmarking.** **(A)** ***Right***: Structural protein profiles (SPPs) per sample type: individual replicas, union and intersection. ***Left***: Zooming on the SPPs of individual replicas and intersection profiles. **(B)** The fold-change of CTCF, RAD21 and SMC3 at domain borders and **(C)** The percentage of identified TADs boundaries occupied by CTCF, RAD21 and SMC3 per sample type in TADpole compared with other 22 TAD callers.


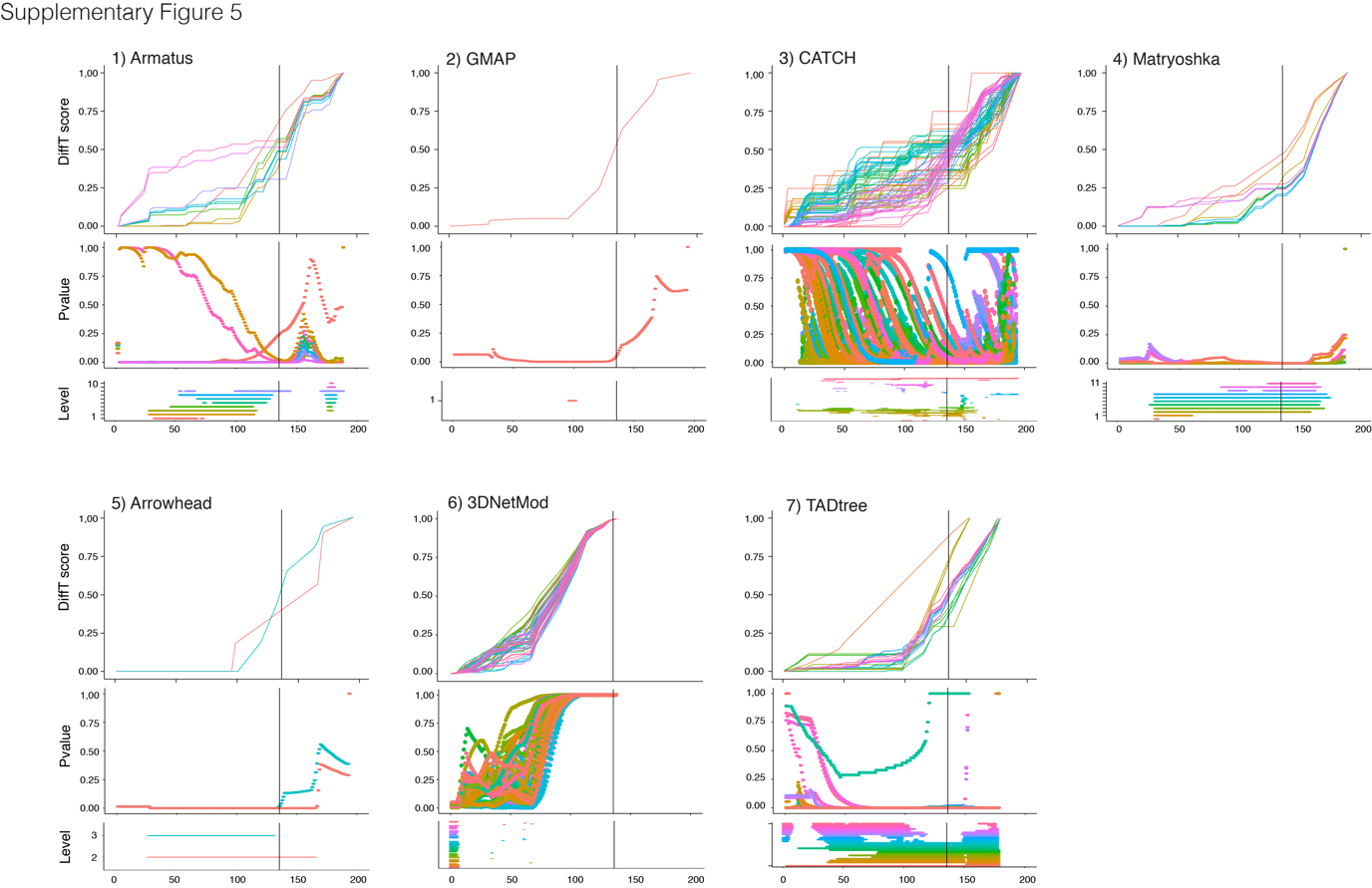


**Supplementary Figure 5. DiffT score profiles across 8 different hierarchical TAD callers.** The DiffT score profiles as a function of the matrix bins for each tool are represented in rows 1 and 4. The p-value profiles per bin for automated detection of significant differences are represented in rows 2 and 5. The bin(s) associated with the minimum p-values per level are represented in rows 3 and 6. Note that only the levels containing at least one bin with a DiffT score associated p-value < 0.05 are shown. In all the panels, the different hierarchical levels recovered by each tool have a distinctive color, while the Inv1 breakpoint is highlighted with a solid black line.

**
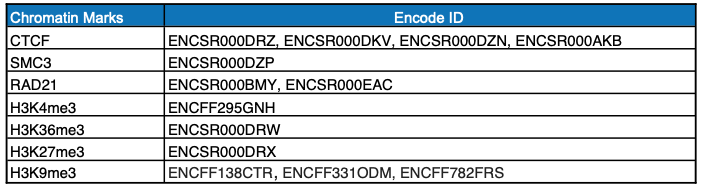
**

**Supplementary Table 1**. Encode IDs of the ChIP-seq experiments used in the biological benchmarking analysis.


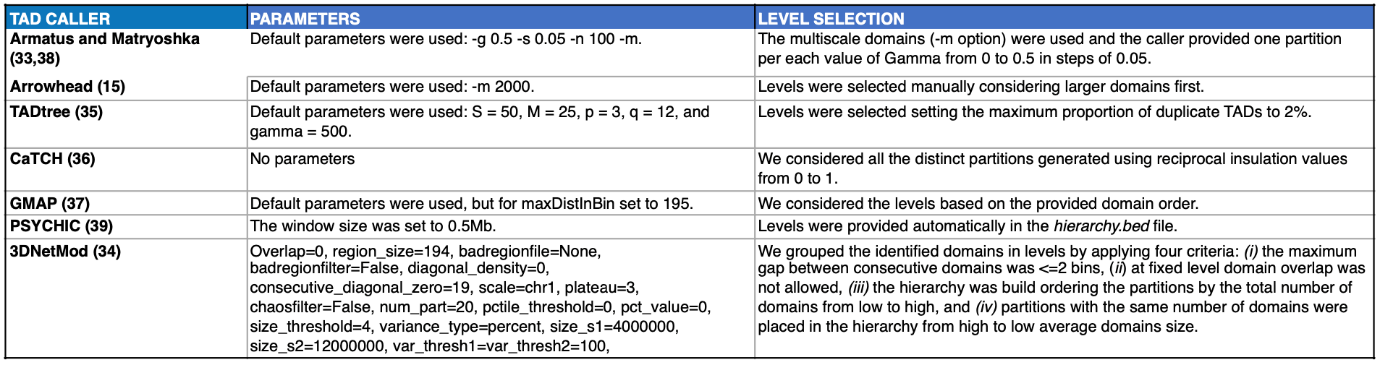


**Supplementary Table 2**. Description of the parameters used and the level selection process followed by each hierarchical TAD caller.


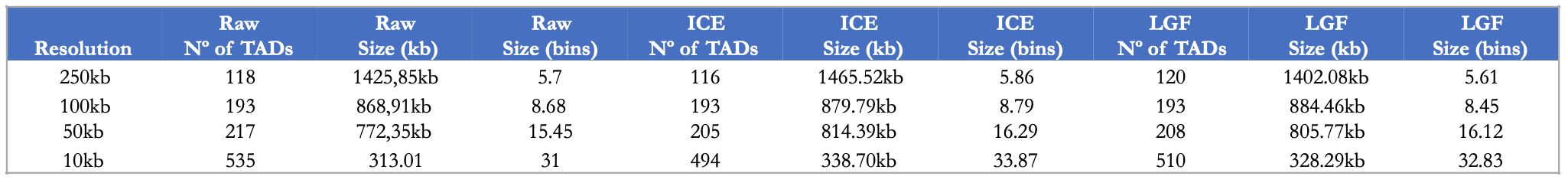


**Supplementary Table 3**. The total number of TADs and the corresponding average size detected in raw and normalized Hi-C matrices (by ICE and LGF) across different resolutions.

**
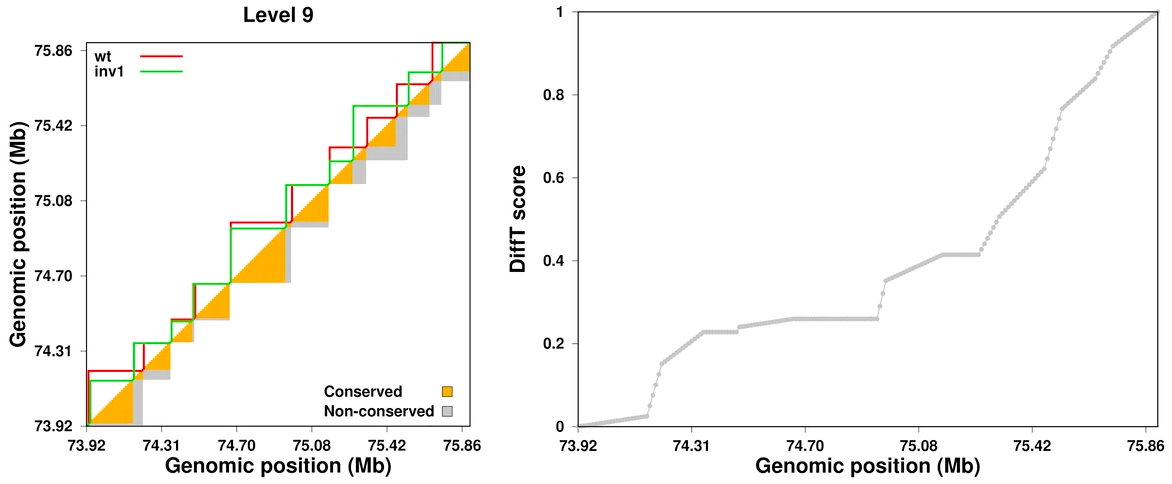
**

**Supplementary video 1. Calculation of the DiffT score for the 9th level of the dendrogram (Figure 4B and C).** The video displays two related synchronized panels. (*Left)* The upper triangle of the matrix shows the TADs borders identified by TADpole in WT and Inv1 matrices as red and green continuous lines, respectively. During the video, the matrix is scanned from the first to the last bin, and simultaneously the lower triangle gets filled with the areas of the TADs that are conserved (in orange) or non-conserved (in gray) between the two partitions. The DiffT score is computed as the normalized sum of the non-conserved (gray) areas. *(Right)* DiffT score profile *versus* the genomic position grows proportional to the gray areas appearing over time in the left panel.
